# Global Temperature Sensing for an Operating Power Transformer Based on Raman Scattering

**DOI:** 10.3390/s20174903

**Published:** 2020-08-30

**Authors:** Yunpeng Liu, Xinye Li, Huan Li, Xiaozhou Fan

**Affiliations:** 1 State Key Laboratory of Alternate Electrical Power System with Renewable Energy Sources, North China Electric Power University, Baoding 071003, China; liuyunpeng@ncepu.edu.cn (Y.L.); lxy@ncepu.edu.cn (X.L.); lih@ncepu.edu.cn (H.L.); 2 Hebei Provincial Key Laboratory of Power Transmission Equipment Security Defence, North China Electric Power University, Baoding 071003, China

**Keywords:** power transformer, condition monitoring, global sensing capability, hotspot location

## Abstract

Traditional monitoring methods cannot obtain the overall thermal information for power transformers. To solve this problem, a distributed fiber optic sensor (DFOS) was creatively applied inside an operating 35 kV power transformer by highly integrating with the electromagnetic wires. Then, the transformer prototype with totally global sensing capability was successfully developed and it was qualified for power grid application through the strict ex-factory tests. The as designed optical fiber sensor works stably all the time with a temperature accuracy of ±0.2 °C and spatial positioning accuracy of 0.8 m. Based on the obtained internal temperature distribution, Gaussian convolution was further applied for the signal processing and hereby, the hotspots for all the windings and iron cores could be accurately traced. The hottest points were located at 89.1% (55 °C) of the high voltage winding height and 89.7% (77.5 °C) of the low voltage winding height. The actual precise hotspot location corrected the traditional cognition on the transformer windings and it would serve as an essential reference for the manufactures. This new nondestructive internal sensing and condition monitoring method also exhibits a promising future for the DFOS applying in the high-voltage electrical apparatus industry.

## 1. Introduction

Transformer overheating is gradually becoming a common problem due to the rapid growth of power consumption, which directly threatens its life expectancy and the safety of the entire power grid. Also, there are increasingly power explosions caused by the long-term imperceptible local overheating. The overheated materials will accelerate the insulation deterioration, cause interturn discharges and result in the final power shutdown. Thus, the internal temperature of a transformer, especially the winding hotspot, is of great practical significance for the transformer healthy operation [1]. However, the current traditional monitoring methods still cannot realize a spatiotemporally continuous temperature detecting, leaving huge monitoring blind area for the transformers [2,3].

Current research on temperature field inside transformers mainly focuses on two aspects: direct measurement [4,5,6] and indirect calculation [7,8,9]. Thermocouples, fluorescent optical fiber sensors and fiber grating sensors (FBG) are usually used for a multi-point sampling in the direct measurements due to its relatively simple and convenient installation, which leads to a strong operability.

The fluorescent fiber sensor is based on the monotonic relationship between the temperature and the fluorescent lifetime of the probe while the FBG works by detecting the wavelength variation of the reflected light caused by the temperature. Both these optical fiber sensors have advantages in their rapid response and strong light signals. Meanwhile, the good insulation properties and anti-electromagnetic interference characteristics further enlarge their applying scope. 

The remarkable achievement was a measurement performed by Kweon on a 154 kV transformer during the temperature-rise test. The fluorescent fiber sensors were placed on the windings in advance for a local temperature measurement [10]. Thereafter, Gong built a temperature measurement system based on FBG (Fiber Bragg Grating) sensor for a 110 kV transformer [11]. Then, Arabul arranged a large quantity of fluorescent fiber sensors in the oil passages between each adjacent winding wire for the detection of overheated regions in a 1.5 MVA power transformer [1,12]. However, the direct measurement method often requires an estimation of the hot spot position in advance, which is highly subjective and empirical, and cannot achieve distributed measurement in the entire region. Thus, it leaves a large monitoring blind area.

In contrast, the indirect calculation, as its name suggests, uses a numerical method to solve the transformer temperature field based on a thermal circuit model or a mathematical analysis. In addition, it can thus theoretically achieve a distributed detection [13]. The outstanding achievement was from Swift, who established the first thermal circuit model for the top-oil and the winding hotspots and the work was also verified by serial corresponding field tests [14]. Then, Susa built a transformer thermal model based on hotspots and the ambient temperature gradients [15,16]. Meanwhile, Jardini improved the aforesaid models by introducing the differential equations and performed an actual test on a 138 kV power transformer [17]. However, the indirect calculation, limited by the convergence of the numerical method, often greatly simplifies the actual conditions to obtain a stable solution. In addition, the different models built for the same transformer usually lead to different results. Hereby, the calculation results can only serve as a reference rather than a substitute of the actual temperature distribution [7]. It is still difficult for traditional methods to obtain the real-time full-region temperature distribution under complex circumstances inside power transformers.

The distributed fiber optic sensing technology, developing rapidly in recent years, has gradually been applied in some super projects such as bridge and tunnel monitoring, petroleum and gas lines monitoring, power transmission lines monitoring, civil engineering, coal mining and so forth. The spatiotemporally continuous detecting advantages furtherly enlarge its application [18,19,20].

Yilmaz has realized the thermal monitoring on 154 kV transmission line based on ROTDR (Raman Optical Time Domain Reflectometry) technology [21]. Liu has accurately located the leakage point in an underground diaphragm wall seepage monitoring through the DFOS [22]. Boujia successfully adopted the DFOS for soil-structure interaction [23]. Gao further extended the application to the transformer winding deformation [24]. Hu proved the feasibility of monitoring the roof strata movement in coal mining by DFOS [25]. Delepine [26] and Jensen [27] realized the distributed temperature measurement of an underground nuclear waste storage and the nuclear reactor cooling circuit, respectively. The DFOS wild application in various fields under different tough conditions exhibits its mature and stable performance and shows great potential in solving the power transformer overheating problems.

In this contribution, an optical fiber composite winding wire has been designed. On this basis, an oil-immersed 35 kV power transformer prototype with built-in distributed optical fiber sensors was successfully fabricated and qualified for power grid operation through the corresponding type tests. Moreover, the real-time temperature inside an operating power transformer was obtained in a spatiotemporally, continuously distributed manner. The hotspot was also located and continuously monitored.

## 2. Detecting Principle

### 2.1. Principle of the DFOS Temperature Sensor Based on Raman Scattering

When propagating in optical fiber, the light will be scattered to different degrees due to the collisions with media molecules, resulting in a scattering spectrum with different frequencies [19]. Among these, Raman scattering has been discovered a strong temperature sensitivity.

The luminous flux of Stokes Raman scattering generated by each light pulse [28] is presented in Equation (1):

(1) $$\Phi_{S}=K_{S}\cdot S\cdot v_{S}^{4}\cdot \phi_{e}\cdot R_{S}(T)\cdot \exp[-(\alpha_{0}+\alpha_{S})\cdot L]$$

The luminous flux of anti-Stokes Raman scattering can be expressed as Equation (2):

(2) $$\Phi_{AS}=K_{AS}\cdot S\cdot v_{AS}^{4}\cdot \phi_{e}\cdot R_{AS}(T)\cdot \exp[-(\alpha_{0}+\alpha_{AS})\cdot L]$$

where *K_S_* and *K_AS_* are the cross-section coefficients of optical fiber related to Stokes scattering and anti-Stokes scattering, respectively; *S* is the backscattering factor of the fiber; *v_s_* and *v_AS_* are the frequencies of Stokes and anti-Stokes scattering photons; *Φ_e_* is the number of incident laser pulse photons; *α*_0_, *α_S_* and *α_AS_* are the average propagation loss factors of incident light, Stokes scattering light and anti-Stokes scattering light, respectively; *L* is the distance between the incident end of fiber and the measured point; and *R_S_(T)* and *R_AS_(T)* are the corresponding coefficients, related to the particle distribution of fiber molecules at different energy levels, which act as the temperature modulation functions of Stokes Raman scattering and anti-Stokes Raman scattering, as shown in Equations (3) and (4).

(3) $$R_{S}(T)={\left[1-\exp(-h\Delta v/kT)\right]}^{-1}$$

(4) $$R_{AS}(T)={\left[\exp(h\Delta v/kT)-1\right]}^{-1}$$

where *h* is the Planck constant (*h* = 6.626 × 10^−34^ J·s); Δ*v* is the Raman phonon frequency (Δ*v* = 1.32 × 10^13^ Hz); *k* is the Boltzmann constant (*k* = 1.38 × 10^−23^ J·K^−1^); and *T* is the thermodynamic temperature.

According to Equations (1)–(4), the function of temperature about the corresponding position can be demodulated. That is to say, the temperature information along the entire optical fiber can be obtained, as shown in Equations (5) and (6).

(5) $$\frac{1}{T}=\frac{1}{T_{0}}-\frac{k}{k\Delta v}\ln(\frac{\Phi_{AS}(T)/\Phi_{S}(T)}{\Phi_{AS}(T_{0})/\Phi_{S}(T_{0})})=\frac{1}{T_{0}}-\frac{k}{k\Delta v}\ln(F(T))$$

(6) $$(F(T))=\frac{\Phi_{AS}(T)/\Phi_{S}(T)}{\Phi_{AS}(T_{0})/\Phi_{S}(T_{0})}=\frac{\exp(-h\Delta v/kT)}{\exp(-h\Delta v/kT_{0})}$$

where *T*_0_ is the temperature of the calibrated fiber.

The temperature distribution along the fiber laying path can be hereby obtained by just measuring out the electrical levels of *Φ_AS_(T)*, *Φ_S_(T)*, *Φ_AS_(T_*0*_)* and *Φ_S_(T_*0*_)* after the photoelectric conversion [29].

**Figure 1:**
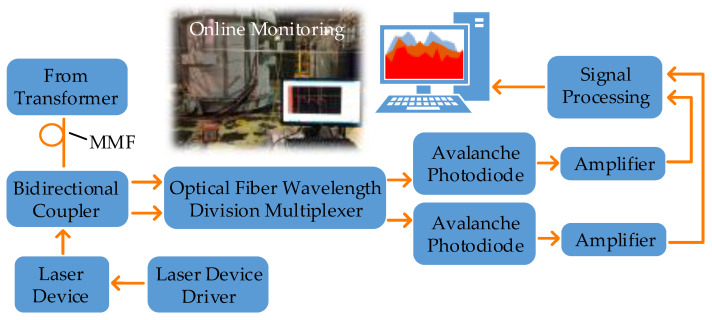
Principle of transformer temperature on-line monitoring based on DFOS.

The working process of the distributed optical fiber temperature sensor is shown in Figure 1.

The pulsed laser enters the fiber through one end of the integrated wavelength division multiplexer (involving a 1 × 2 bidirectional coupler (BDC) and an optical wavelength division multiplexer (OWDM)). Then its backscattering will be divided into Stokes and anti-Stokes Raman light. After the photoelectric conversion in avalanche photodiode (APD) and accumulation of analog-to-digital conversion, the processed signal will be delivered to a computer for temperature demodulation and data storage to achieve online distributed temperature measurement [30].

### 2.2. Pricinple of Signal Denoising Based on Gaussian Convolution

Gaussian convolution is an effective denoising method which is usually applied for image processing. However, there are also scholars who have successfully used this method to suppress the noises generated from optical fibers. Myonghwan performed a unidirectional convolution on the FBG temperature sensors to get a more accurate result [31]. In this paper, a two-dimensional Gaussian distribution will be used, as exhibited in Equation (7).

(7) $$G(x,y)=\frac{1}{2\pi \sigma^{2}}e^{\frac{-x^{2}-y^{2}}{2\sigma^{2}}}=\frac{1}{2\pi \sigma^{2}}e^{-\frac{x^{2}}{2\sigma^{2}}}*\frac{1}{2\pi \sigma^{2}}e^{-\frac{y^{2}}{2\sigma^{2}}}=G(x)*G(y)$$

where *x, y* are the data coordinates in spatial domain; *σ* is the standard deviation of the sensing errors; *G (x, y)* is the Gaussian function.

The Fourier transform of Gaussian function remains the same, and hereby it can form a low-pass filter with smooth performance in frequency domain. The transfer function of a Gaussian low-pass filter in frequency domain can be described as Equation (8).

(8) $$H(u,v)=e^{-D^{2}(u,v)/2D_{0}^{2}}$$

where *u, v* are the Fourier transforms of *x, y* in frequency domain; *D* is the distance from the point *(u, v)* to the origin of the frequency rectangle; *D*_0_ is the cut-off frequency.

## 3. Fabrication and Platform Setup

### 3.1. Pre-Experiments

To ensure that the optical fiber works stably under the high temperature environment of transformer and has good compatibility with transformer oil, in our former series work [32], the safety test was performed through the accelerated thermal aging method (130 °C for 576 h). Tetrafluoroethylene (ETFE) and polyimide (PI) was finally selected as the optical fiber sheath and coating layer material due to their stable performance after the long-term aging process in the transformer oil. Meanwhile, the electric performance of the selected optical fiber was also qualified for the actual operating of a transformer due to its good insulation properties.

### 3.2. Manufacture of the DFOS Integrated Coil

**Figure 2:**
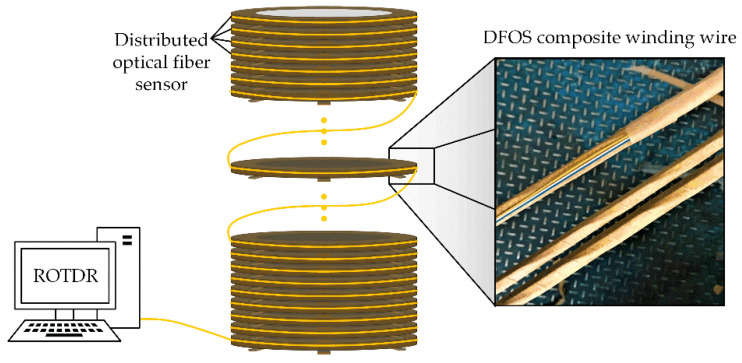
Schematic diagram of distributed sensing optical fiber composite winding.

The optical fiber contains no metal materials and thus has no influence on the normal operation of transformers. To get the full-region temperature distribution inside power transformers, the distributed optical fiber sensor was embedded into the outermost turn of winding wire and winded synchronously with the wire during the normal manufacturing process, as shown in Figure 2. Meanwhile, a layer of insulating paper was used to wrap the optical fiber composite wire for reducing the effect of possible vibrations and knocks during the manufacturing of transformers. The as designed fiber integrated electromagnetic wire completely maintains the original winding structure. During the normal operation of transformer, the sensor will be synchronously heated with the adjacent wire. Hereby, it can easily obtain the real-time temperature along the whole winding by just detecting the changes in Raman scattering signal. The optical fiber laying process has no interruption to the normal manufacturing of a power transformer and enjoys a strong universality. In addition, the pulsed laser signal will not cause any electromagnetic interference inside transformers.

**Figure 3:**
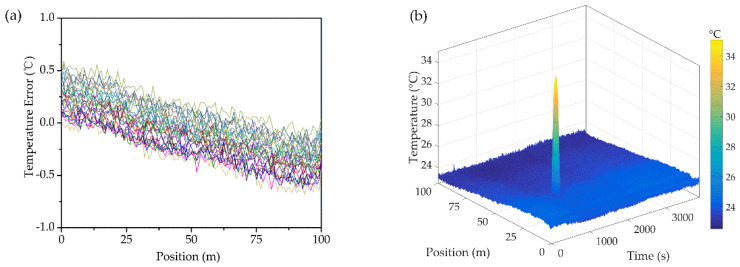
(**a**) Temperature measurement error of the as designed fiber sensor (**b**) Temperature measurement sensitivity of the hotspot.

To furtherly test the positioning accuracy of the aforesaid fiber composite coil, a 2 m length of heating tape (acting as a local hotspot) was closely attached to the inner side of the winding wire while the remaining region was at ambient temperature (23–24 °C). The heating tape was controlled by the muti-point sampling of thermocouples with a temperature accuracy of 0.1 °C. The hotspot temperature measured by thermocouples is 35.2 °C (in average) compared to 35.7 °C detected by DFOS. The result, exhibited in Figure 3b, has shown that the sensing fiber was rather sensitive to the local overheating and has a spatial accuracy of 0.8 m (which is sufficient to locate the exact overheated winding turn). The temperature measuring error was less than 1 °C (shown in Figure 3a). The fluctuations along the sensing fiber, possibly caused by the detecting equipment, could be furtherly smoothed through algorithms.

### 3.3. Online Monitoring Platform Setup

To furtherly extend the monitoring range inside power transformers, the distributed optical fiber sensor was also uniformly winded along the core limbs. In addition, the optical fiber feedthroughs were installed on a flange sealed on the oil tank. 

**Figure 4:**
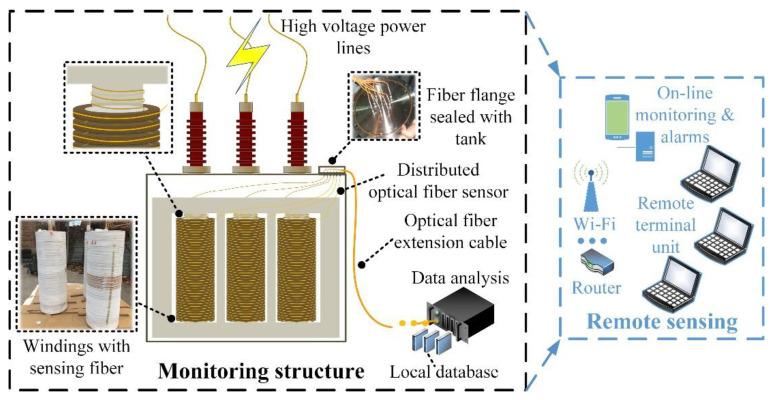
35 kV distributed optical fiber sensor integrated power transformer sensing structure.

The optical fiber composite transformer prototype was fabricated in strict accordance with the transformer manufacturing process, followed by ex-factory type tests based on relevant industrial standards of IEC 60076 [33]. The product was qualified to put into power grid operation after performing serial tests including load loss and no-load loss measurement, temperature-rise test, induced over voltage withstand test, power-frequency voltage withstand test, tightness test, dielectric routine tests and so forth. The field online monitoring platform is exhibited in Figure 4. In addition, the specific parameters of the studied power transformer are listed in Table 1.

A total length of around 1029 m distributed optical fiber sensor was applied inside the transformer for a full-region temperature online monitoring. In addition, the temperature measurement range of the optical fiber sensor was −30–270 °C. The detecting equipment is a commercial ROTDR product (BY-DTS-4020, Weihai Beiyang Optoelectronic Info-Tech Co. Ltd., Weihai, China) and the relative parameters of the DFOS and the instrument are listed in Table 2. The common temperature sensors applied for the power transformers are also listed below for comparison.

**Figure 5:**
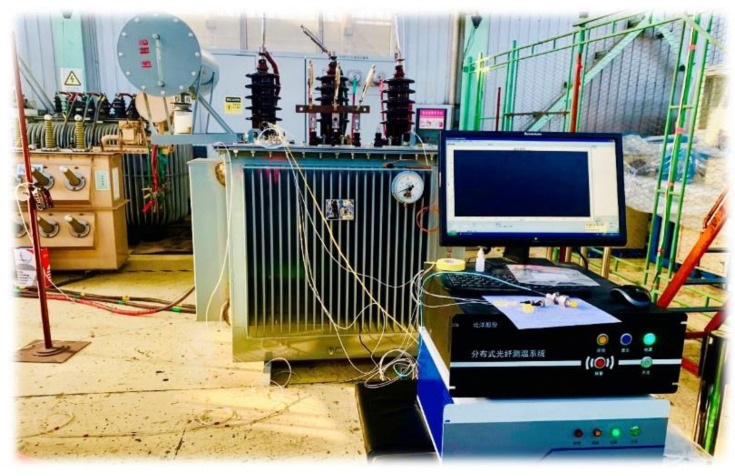
Field temperature online monitoring.

The optical fiber, integrated with the winding wire, was connected to a data-analysis equipment through a fiber flange and the collected real-time information was delivered to a remote monitoring unit through wireless transmission. The field online monitoring platform was exhibited in Figure 5.

## 4. Detecting Results 

### 4.1. Denoising of the Sensing Fiber

**Figure 6:**
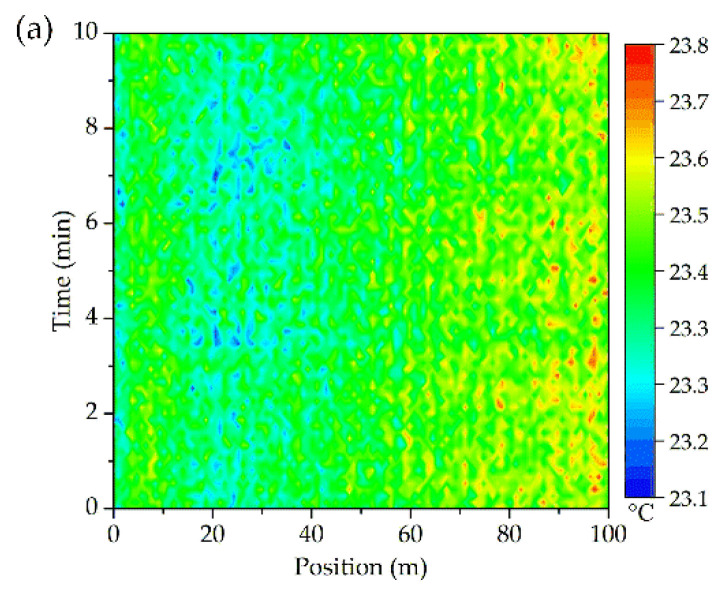

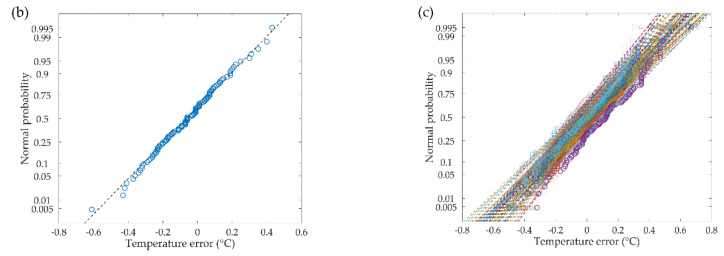
(**a**) Temperature noises; and probability plot for normal distribution of the sensing errors; (**b**) At 1 min; (**c**) Throughout the whole time.

Before the experiments, the ROTDR product was calibrated through a high accuracy thermostatic cabinet with the temperature controlling error less than 0.01 °C. In addition, ambient temperature (also taken as the initial temperature for further noises processing) was measured by both the thermocouples (23.4 °C on average) and the fiber (shown in Figure 6a). It shows that the DFOS is rather stable and accurate. Though the detecting noises of the optical fiber is inevitable, they can greatly affect the accurate location of the hotspots. Thus, data cleaning work needs to be done in advance. The first 100 m sensing fiber was selected for the convenience of analysis and the remaining shared a similar distribution.

For the one continuous sensing fiber, the detecting noises (at different times) were compared with the normal distribution and the probability plots were exhibited in Figure 6b,c. Almost all the sensing errors matched well with the normal distribution and the asymptotic significance (2-tailed) was above 0.94 according to the Kolmogorov-Smirnov test.

**Figure 7:**
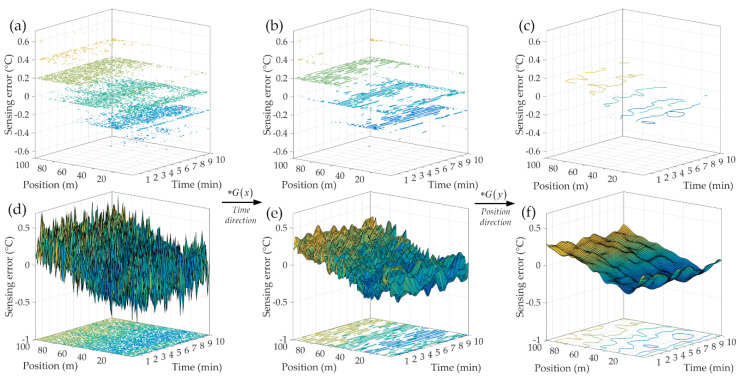
Sensing error contour every 0.2 °C (**a**,**d**) raw data; (**b**,**e**) Gaussian convolution along Time direction; (**c**,**f**) Gaussian convolution along Position direction.

Thus, based on the normal distribution properties of the sensing error, Gaussian convolution method was applied to eliminate the background noises. A 7 × 7 discretization sliding window (*σ* = *D_0_* = 1) was established for the convolution operation. The detecting results before and after the Gaussian convolution are shown in Figure 7.

As can be seen from Figure 7, the sensing background noises were greatly suppressed (the detecting accuracy has been improved to 0.2 °C). In addition, this convolution method can be hereby used for the following experiments.

### 4.2. Detecing Results and Discussion

**Figure 8:**
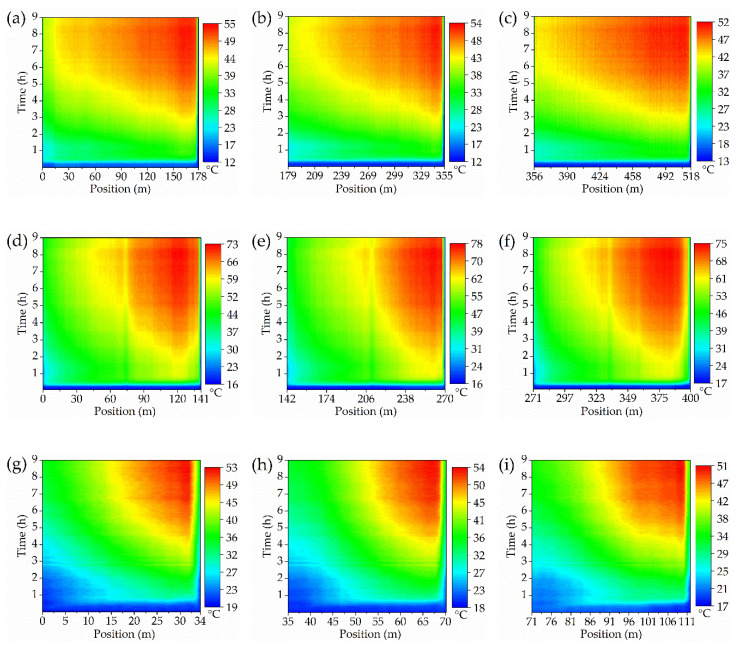
Full time temperature distribution along the (**a**) HV winding, phase A; (**b**) HV winding, phase B; (**c**) HV winding, phase C; (**d**) LV winding, phase A; (**e**) LV winding, phase B; (**f**) LV winding, phase C; (**g**) Core limb, phase A; (**h**) Core limb, phase B; (**i**) Core limb, phase C.

The spatiotemporal temperature of full regions inside the operating transformer was monitored in a distributed manner, as exhibited in Figure 8. The as designed optical fiber sensor displays an effective sensing performance under the complex thermal conditions inside the power transformer and works stably all the time. The actual temperature distribution inside power transformer exhibits a strong position dependence, attributed to the different surrounding circumstances, such as irregular oil flow, various structural components, etc. Thus, the point-type detecting method inevitably exists huge blind zones, leaving hidden dangers for the safe operation.

The fiber laying length of each monitoring area is also exhibited in Figure 8. For all the windings and the three phase core limbs, the sensing fiber was connected with each other through the optical fiber patch cord on the outer side of the fiber flange (the detected data of these extra optical fibers have not been included into the results).

**Figure 9:**
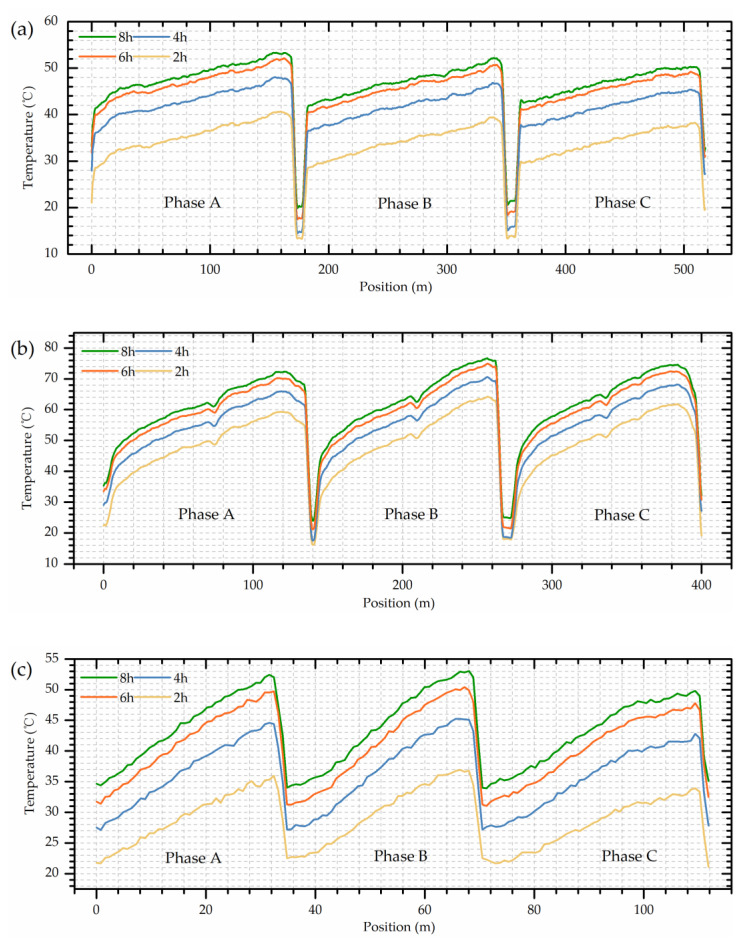
Spatial temperature at 2 h, 4 h, 6 h, and 8 h of (**a**) HV winding; (**b**) LV winding; (**c**) Iron core.

The heat-run test was performed with short-circuit method, lasting for nearly 9 h, was composed of two steps, namely applying total losses (first 8 h) and applying rated current (the last hour). The detailed temperature distribution is shown in Figure 9.

As for Figure 9a, the temperature of HV winding presents an increasing trend with the sensing fiber length (or the winding wire) and the hotspots tend to appear near the top of the winding. Interestingly, a higher winding temperature was observed in phase A compared with the other two phases, which might be due to the manufacturing deviations, internal structure changes or the irregular oil flows. In general, the three phases windings maintain a similar temperature distribution. The highest temperature around 55 °C (at 8.3 h) during the whole test was located at 158.6 m of the phase A winding (89.1% of the winding height).

By contrast, the LV winding displays a relatively higher temperature (due to its higher rated current), as shown in Figure 9b. However, there exists a temperature drop at the top of the winding (around 6–8 °C), especially evident in phase A and C. This new phenomenon is quite different from the traditional cognition about the transformer winding which believes the temperature is always higher in the top area and the temperature distribution should exhibit a monotonically increasing trend with the winding height (according to the International Electrotechnical Commission (IEC) [33]). However, the traditional conjecture was based on the point-type measurements such as thermocouples or fiber gratings, and they lack in global sensing capability. Thus, the first detailed internal data have revealed a very interesting pattern that the hotspot is not located at the winding top as is often supposed, but tends to appear at about 90% of the winding height. This might be due to the relatively good heat dissipation conditions in the top area which is usually ignored by most people. The highest temperature of LV winding was 77.5 °C, located at 89.7% of phase B winding height. The little temperature drop in the middle of LV windings may be caused by the relatively wide oil passages (winding structure). In addition, the significant difference between HV and LV windings may be due to the different relative positions and different load currents.

The iron core limb temperature, displayed in Figure 9c, also shows a positive correlation with the height (the optical fiber was uniformly and spirally winded along the limb) and the highest temperature was around 54 °C. However, the iron core was composed of many layers of laminates and its structure was completely different from the windings, so the temperature distribution could be different.

The detailed hotspots temperature and their corresponding locations are listed in Table 3, Table 4 and Table 5 during the whole heat-run test.

As can be seen from Table 3 and Table 4, the hotspots of HV windings fluctuated between 88–92% of the winding height and those of the LV windings were located between 84–90% of the height. The actual detected data indicates that there exists an obvious temperature drop in the winding top area and the hotspot location should be much lower than the commonly believed, especially lower than the assumption proposed by International Electrotechnical Commission (IEC) in 2011 and 2018 (International standard of IEC 60076-2 and IEC 60076-7, in which the internal temperature detecting of a power transformer is still based on the point-type optical fiber measurements) [33,34].

**Figure 10:**
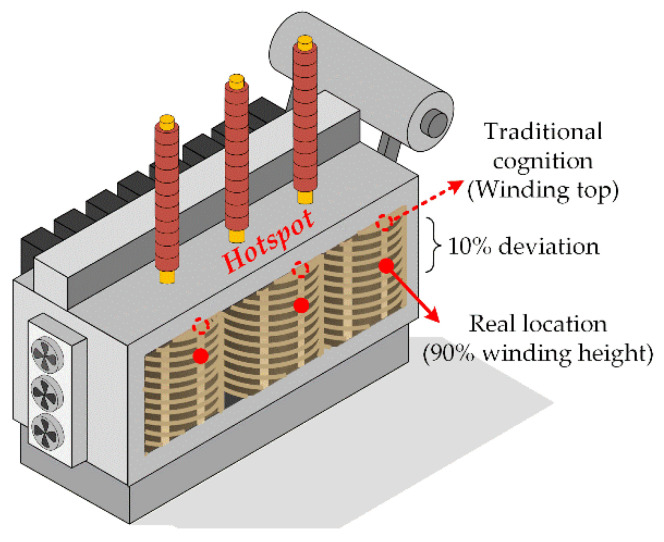
Hotspot real location compared to the traditional cognition.

A long-standing cognition believes that the hotspot always appears at the top of windings. However, this newly observed phenomenon obviously breaks this traditional cognition (Figure 10). It exists throughout the entire experiment and is not by chance. The transformer prototype was fabricated in strict accordance with the manufacturing process and a second heat-run test showed no more than 2% difference in results. Meanwhile, it is also supported by a relevant numerical simulation study on the oil-immersed power transformer conducted by Liu in 2019 (in which the author also discovered a temperature drop at around 90% of the winding height but lacked the actual data support) [13]. Taghikhani also noticed this phenomenon and gave its theoretical foundation according to heat transfer theories [35].

However, there are still lacking field research about the distributed internal temperature of an operating power transformer due to the limitation of the traditional point-type detecting methods. In addition, the new discovery in this paper will undoubtedly correct the conventional thoughts.

In a word, the hotspot of an operating oil-immersed power transformer is very likely located at around 90% of the winding height according to the field distributed temperature monitoring. In addition, extra protection of this area is thus in urgent need, especially in the thermal and insulation aspects. 

### 4.3. Comparison with IEC Models

**Figure 11:**
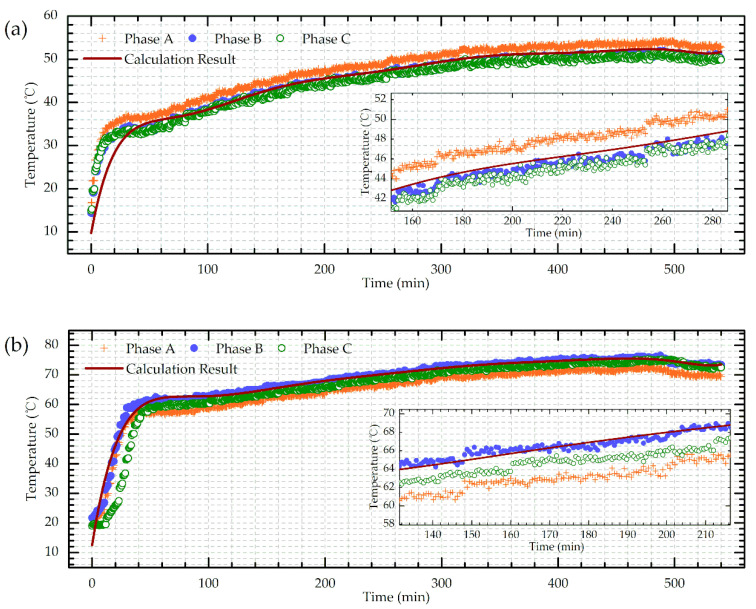
Hotspot temperature transient process of (**a**) HV winding; (**b**) LV winding.

The detected hotspot temperature changes in time domain were also compared with the IEC standard calculation model, as shown in Figure 11 (90%/87% of the HV/LV winding height was selected as the average hotspot location). Three phases exhibit a similar tendency but with a little difference possibly caused by the deviations in the manufacturing process.

As can be seen from Figure 11, the hotspots of all the windings came to a relatively steady state at around 1h and gradually rose to the final temperature, which corresponds well with the theoretical calculation results. The hotspot calculation was established on a thermal circuit model where all the transformer components were equivalent to the corresponding thermal capacitors, thermal resistances, etc. The hotspot temperature ((Equation (9)) can be obtained as the sum of top-oil temperature (Equation (10)) and the hotspot temperature rise (Equation (11)). The detailed calculating process was exhibited as follows.

(9) $$\theta_{h}(t)=\theta_{o}(t)+\Delta \theta_{h}(t)$$

(10) $$\theta_{o}(t)=\theta_{a}(t)+\Delta \theta_{oi}+[\Delta \theta_{or}\times {\left(\frac{1+R\times K^{2}}{1+R}\right)}^{x}-\Delta \theta_{oi}]\times [1-e^{\left(-t)/(k_{11}\times \tau_{o}\right)}]$$

(11) $$\Delta \theta_{h}(t)=\Delta \theta_{h1}(t)-\Delta \theta_{h2}(t)$$

where *θ_h_(t)* is the hotspot temperature, °C; *θ_o_(t)* is the top-oil temperature, °C; Δ*θ_h_(t)* is the hotspot to top-oil gradient, °C; *θ_a_(t)* is the ambient temperature, °C; Δ*θ_oi_* is the top-oil temperature rise at start, °C; Δ*θ_or_* is the top-oil temperature rise in steady state at rated losses, °C; *R* is the ratio of load losses at rated current to no-load losses at rated voltage, equal to 7.67 for this transformer; *K* is the load factor (load current/rated current), decreasing from 1.19 (0–1 h) to 1.06 (1–8 h) and finally to 1.0 (8–9 h); *k_11_* is the thermal model constant, taken for 1.0 in this study; *τ_o_* (min) is the oil time constant, considered to be 180; *x* is the oil exponent, taken for 0.8 in an ONAN transformer.

The parameters, Δ*θ_h1_(t)* and Δ*θ_h2_(t)*, can be solved from the two differential equations, as shown in Equations (12) and (13).

(12) $$\frac{d\Delta \theta_{h1}(t)}{dt}=[k_{21}K^{y}\Delta \theta_{hr}-\Delta \theta_{h1}(t)]\times {\left(k_{22}\tau_{w}\right)}^{-1}$$

(13) $$\frac{d\Delta \theta_{h2}(t)}{dt}=[(k_{21}-1)K^{y}\Delta \theta_{hr}-\Delta \theta_{h2}(t)]\times \left(k_{22}/\tau_{o}\right)$$

where *k*_21_, *k*_22_ are the thermal model constant, taken for 1.0 and 2.0 respectively; *y* is the winding exponent, taken for 1.6 in this transformer; Δ*θ_hr_* is the hotspot to top-oil gradient at rated current, °C; *τ_w_* is the winding time constant, considered to be 4 min in the calculation.

The DFOS measured results exhibit a good consistency with the calculation results in the hotspot time domain, proving the effective monitoring ability of the designed sensing scheme. However, the calculating method cannot get the internal temperature feedback in real time due to the fact that the load factor *K* actually would continuously change during the normal operation instead of holding a constant value (usually the power load could vary hugely even in one minute). Thus, it is unrealistic to dynamically update the computations. Meanwhile, the corresponding transformer parameters could also imperceptibly change during its service life, leading to inevitable deviations in the hotspot temperature prediction. Moreover, the calculating model still cannot detect the accurate location of the hotspots and is lack of the global temperature sensing capability. All in all, although the theoretical calculation matched well with the DFOS detecting results in a temperature-rise test, it is still hard to meet the field online real-time monitoring requirements.

## 5. Conclusions

The DFOS composite power transformer prototype has been successfully developed and put into actual online operation through a heat-run test. The designed sensing scheme has realized a global internal temperature detecting and works effectively all the time. The hotspots of all the windings and core limbs are accurately located, which has greatly changed the conventional cognitions. The following conclusions can be drawn:

- The distributed fiber optic sensor integrated windings can serve as an effective role in the global sensing of an operating power transformer with a temperature accuracy of ±0.2 °C and spatial accuracy of 0.8 m (one turn of the windings).
- The temperature sensing error along one continuous optical fiber is highly consistent with the normal distribution, which indicates that the noises can be greatly suppressed through the Gaussian convolution and hence, the detecting accuracy can be further improved.
- This work is the first to reveal the global internal temperature distribution of an operating power transformer and the detailed thermal information will serve as an important reference for the relevant scholars, especially for the manufacturers.
- The actual temperature distribution of both the HV and LV windings exhibits a decline tendency in the top area of the winding, which means that the real location of hotspot should be much lower than the traditional cognition (which believes it always appears at the winding top). In addition, 90% of the winding height is recommended as the precise hotspot location in this paper according to the real detected data. Further extra protection is needed in this region, especially in thermal and insulation aspects.

## Figures and Tables

**Table 1:** Specifications of the transformer.

Classification	Specification
Rated voltage/V	35,000/400
Rated current/A	3.3/288.7
Rated capacity/kVA	200, 50 Hz
Core type	Shell
Temperature rise limit/°C	Top oil: 60
	Average winding: 65
Cooling method	ONAN

**Table 2:** Comparison between DFOS and other common temperature sensors for the transformer.

Classification	Distributed	Point-Type ^1^		
	DFOS	Fiber Bragg Grating	Fluorescence Fiber	Thermocouple ^2^
Range/°C	−30–270	−40–120	−30–200	−100–1300
Accuracy/°C	1.0	1.0	1.0	0.75% T
Resolution/°C	0.1	0.1	0.1	0.1
Response time/s	2–10	1	1	1
Sensor type ^3^/mm	Fiber: *Φ* 0.9	Probe: (*Φ* 8 × *L* 70)	Probe: (*Φ* 10 × *L* 45)	Thermode: (*Φ* 4)
Durability ^4^/year	20	20	20	3–5
Cost ^5^	Moderate	Low	Low	Very cheap
Detecting length/m	0–2000	None	None	None

^1^ Average parameters for common commercial products. ^2^ K-type thermocouple (most commonly used). ^3^ *Φ* means diameter and *L* means length. ^4^ On average and under normal situations. However, the actual life expectancy is up to the specific use environment. Normally, the optical fiber lifetime is up to the sheath material (usually designed for about 20 years). ^5^ Mainly reflects in the measuring equipment (the cost of optical fiber is same and rather low).

**Table 3:** The hotspots information of HV winding during the operating time.

Time/h	Phase A		Phase B		Phase C	
	Temp./°C	Pos./%	Temp./°C	Pos./%	Temp./°C	Pos./%
2	40.9	89.8	39.5	89.5	38.3	91.9
4	48.0	88.2	46.8	90.9	45.4	91.5
6	52.4	91.1	50.7	91.8	49.3	91.3
8	53.5	91.0	52.6	91.3	50.5	91.7

**Table 4:** The hotspots information of LV winding during the operating time.

Time/h	Phase A		Phase B		Phase C	
	Temp./°C	Pos./%	Temp./°C	Pos./%	Temp./°C	Pos./%
2	59.4	84.4	64.3	89.5	61.9	87.5
4	65.9	84.9	70.6	89.7	68.3	87.7
6	70.3	84.1	74.9	89.6	72.4	87.6
8	72.5	85.4	77.5	89.7	74.6	87.7

**Table 5:** The hotspots information of iron core limb during the operating time.

Time/h	Phase A		Phase B		Phase C	
	Temp./°C	Pos./%	Temp./°C	Pos./%	Temp./°C	Pos./%
2	36.0	92.9	36.8	94.1	33.9	95.9
4	44.6	95.3	45.2	93.4	42.8	96.0
6	49.7	92.9	50.4	92.3	47.8	95.9
8	52.4	95.3	53.1	94.4	49.8	95.9
